# Translation and interpreting self-efficacy: a review of published measures

**DOI:** 10.3389/fpsyg.2025.1632366

**Published:** 2025-10-01

**Authors:** Muhammad M. M. Abdel Latif, Majed M. Alhamad

**Affiliations:** ^1^Faculty of Graduate Studies of Education, Cairo University, Giza, Egypt; ^2^Department of Studies of Arabic (L2), College of Language Sciences, King Saud University, 11652 Riyadh, Saudi Arabia

**Keywords:** translation self-efficacy, interpreting self-efficacy, self-efficacy measures, translation motivation, translator training

## Abstract

An important area of research in translation and interpreting assessment focuses on measuring translators’ and interpreters’ self-ability beliefs, normally characterized by two main constructs: self-efficacy and self-concept. Self-ability beliefs have a potential relationship with translators’ and interpreters’ task performance and perseverance. While several self-efficacy measures for translators and interpreters exist, no self-concept scales appear to have been published to date. In this article, we review seven published scales assessing translation and interpreting self-efficacy (four translation and three interpreting measures). In our review, we discuss the extent to which the items of the scales represent the constructs being assessed, as well as the translation or interpreting competences they cover. The review indicated that the seven self-efficacy measures focus on only a limited number of translation and interpreting competences. In addition, their items assess the target competences only partially. A few items related to self-concept were also found in the three reviewed interpreting self-efficacy measures. It is concluded that advancing translation and interpreting self-efficacy measurements requires adopting different orientations. The article highlights the self-efficacy measurement gaps that should be addressed in future translation and interpreting research.

## 1 Introduction

Individuals’ self-beliefs are closely associated with their thinking and functioning (Pajares and Schunk, 2002). According to Bandura (1986), individuals have a self-system that motivates them to exercise control over their thoughts, motives, and behaviors. Those with high self-ability levels are well-prepared to educate themselves when they have to depend on their own learning initiatives (Bandura, 1986). Two main self-belief constructs have been identified in the psychological literature: self-efficacy and self-concept.

Self-efficacy can be broadly defined as an individual’s beliefs about their ability to demonstrate specific skills when performing a given task. Self-concept is a construct closely related to self-efficacy (Zimmerman, 2000; Bong and Clark, 1999), and it refers to an individual’s beliefs about their general ability, giftedness, and capacity to learn in a particular domain. In other words, self-efficacy is a task-specific perceived ability construct, while self-concept is a general one. In their clarification of the difference between the two contracts, Bong and Skaalvik (2003) provided the example of a person’s perceived ability to achieve a 6-foot high jump—that is, jumping a particular height under certain conditions—as a self-efficacy judgment, in contrast to their general belief about their competence in high jumping, which represents a self-concept judgment. Therefore, it is ‘judgment specificity’ that mainly distinguishes between the two constructs (Ferla et al., 2009, p. 500). Ferla et al. (2009) concluded that “self-concept represents a more past-oriented, aggregated and relatively stable judgment about one’s self-perceived ability in a particular [area, but] self-efficacy represents a context-specific and relatively future-oriented judgment about one’s confidence for successfully performing an upcoming … task” (p. 502). Due to the differentiating and complementary roles of these two constructs, it is important to review the advancements made in their measurements in various fields.

Both self-efficacy and self-concept have been heavily researched in some language areas. For example, the two constructs have received considerable attention in writing studies since the turn of the century. This has been reflected in the publication of more than 20 writing self-efficacy scales and six additional scales measuring writing self-concept (Abdel Latif, 2019, 2021). Similarly, they have been given comparable attention in reading research (e.g., Conradi et al., 2014; Unrau et al., 2018). In research concerned with translation and interpreting—converting a source text into a target text in a written or oral form—the picture has been different (Abdel Latif, 2018, 2020). Although there has been some writing on translators’ and interpreters’ self-concept (del Mar Haro-Soler, 2018; Hunziker Heeb, 2016; Kiraly, 1995), no measures of the constructs seem to have been published so far. Meanwhile, only a few translation and interpreting self-efficacy measures have been developed. An important step in advancing research on translators’ and interpreters’ self-efficacy beliefs is reviewing and evaluating their published measures. On the one hand, this may help in identifying the strengths and weaknesses of the existing scales. On the other hand, it could provide important implications related to nurturing translators’ and interpreters’ self-ability beliefs. Given this, in the present article, we review the published translation and interpreting self-efficacy measures.

## 2 Literature review

### 2.1 The nature of translation and interpreting self-efficacy

As a psychological construct, self-efficacy can be conceptualized as having some common and domain-specific ability belief dimensions. Regarding the common ability belief dimensions of self-efficacy, Bandura (1997) identifies three main ones: (a) magnitude: the perceived difficulty level found in performing a given task; (b) strength: one’s confidence or perceived ability to perform a given task and their persistence when encountering frustrations; and (c) generality: the extent to which efficacy beliefs can be generalized in other similar situations. These three essential dimensions are to be considered when developing self-efficacy measures. Therefore, translation and interpreting self-efficacy constructs share a common focus on individuals’ perceived ability to convert a source text into a target text. For example, Lee (2014) defines interpreting self-efficacy as “a person’s belief about their interpreting competence” (p. 183), while Bolaños-Medina and Núñez’s (2018) describe translation self-efficacy as “individuals’ beliefs in their competence to successfully perform the courses of action needed to produce an acceptable translation” (p. 55). On the other hand, the domain-specific dimensions of self-efficacy vary from one area to another. In writing, for instance, self-efficacy has been conceptualized as encompassing writers’ perceived ability to produce specific written text features, use particular text composing processes or strategies and regulate them, and complete certain writing learning activities. Previous studies have generally focused on one or more of these dimensions (for a comprehensive review, see Abdel Latif, 2021).

With this domain specificity, translation self-efficacy measures are also expected to focus on different competences as compared to interpreting self-efficacy measures. Similarly, the specific competence dimensions addressed in oral interpreting scales are expected to be different from those in sign interpreting self-efficacy scales. While the competences specific to each text conversion field should be considered prior to developing self-efficacy measures, translation competence models have provided some common grounds regarding the competences that translators and interpreters are expected to demonstrate.

### 2.2 Translation/interpreting competence models

Any interpreting or translation competence model aims to provide a framework outlining the *competences or skills, abilities, and knowledge that interpreters and translators should* acquire. Despite their terminological limitation (i.e., translation competences), these models address the competences of both translators and interpreters. Translation competence models have been increasingly published since the turn of the 21st century. For example, PACTE—a research group interested in the Process of Acquisition of Translation Competence and Evaluation—has developed multiple versions of their competence models. PACTE’s (2000) model includes the following competences: transfer competence (converting the source text to meet task requirements), communicative competence, extra-linguistic competence (i.e., general and specialized knowledge types), instrumental-professional (trade and profession skills) competence, psycho-physiological competence (psychomotor, cognitive, and motivational regulation), and strategic competence. PACTE’s (2003) model encompasses bilingual, extralinguistic, psycho-physiological, translation knowledge, instrumental, and strategic competences.

Some other models have introduced different competences. Göpferich (2009), for instance, developed a model encompassing competences related to translators’ communicative ability in both the source and target languages, domain knowledge, research skills, and translation routine activation, as well as psycho-motor and strategic abilities. The model proposed by Schäffner and Adab (2000) includes linguistic, cultural, textual (awareness of textual conventions), domain/subject, research, and transfer competences. Beeby (2011) also proposed a model with the following translation competences: transfer competence, contrastive linguistic competence, contrastive discourse competence, and extra-linguistic competence (i.e., pragmatic, semiotic, and cultural awareness). Eser (2015) provided a more recent model, which includes bilingual, strategic, cultural, domain, textual, research and technological awareness, translation knowledge, and translation service provision competences.

As noted in the above models, both translation and interpreting require possessing a large number of competences. Within each of these competences, there are sub-competences. For example, in their description of language competence in interpreting, Gentile et al. (1996, p. 66) listed interpreters’ abilities to produce a variety of synonyms and registers, use domain-specific expressions, appropriately combine the source text’s verbal and non-verbal cues into the target text, identify and make use of languages rhythm and tone patterns to maximize interpreting efficiency, and easily analyze communicative utterances to anticipate argument presentation and direction. For the effective identification of training needs, translators’ and interpreters’ self-efficacy measures should encompass the competences outlined in the above models, as well as other relevant competences. Intuitively, no single self-efficacy measure will cover all the above-mentioned competences; therefore, we need a variety of measures designed for assessing different competence dimensions.

Apart from covering multiple competencies, there is a need for standardizing the process of developing interpreting and translation self-efficacy measures. As Lee (2014) pointed out, “self-efficacy scale development requires an arduous process involving conceptual analysis of the domain in question, drafting the instrument, and statistical analysis of data” (p. 186). Scale validation also requires ensuring two crucial psychometric properties: validity and reliability. The latter refers to the consistency of the measure’s results over time and under similar conditions. As for validity, it refers to the accuracy of the measure and the degree to which it represents the variable being assessed. Scale validity is a multifaceted concept with different types, including the following: (a) face validity: a validity type conducted during the preliminary stage of measure development to initially check the appropriateness of items and how far they appear to assess the target construct; (b) content validity: another initial validity type used for scrutinizing how the items of the scale fully represent the construct, and it is normally based on expert judgment; (c) construct validity: the extent to which a measure accurately reflects the theoretical construct assessed, and the relationships between the items and the latent factors explaining them (normally through statistical methods such as factor analysis); and (d) criterion-related validity: a validity type for identifying the relationship between the scores of the measure and an external criterion related to how the scale predicts future performance (i.e., predictive validity) or how well it aligns with the measures of close constructs (concurrent validity) (for more detailed information see Kimberlin and Winterstein, 2008; Lim, 2024; Zait and Bertea, 2013). In the present article, we provide a critical review of the validation issues in published translation and interpreting self-efficacy measures.

## 3 The present review: purpose and selection of studies

In our review attempt, we aimed to evaluate the published measures in terms of their relevance to and coverage of translation and interpreting competence dimensions. Therefore, we tried to answer the following two research questions:

- How representative are the items of the published translation and interpreting self-efficacy measures in assessing the target competences?
- To what extent are translation and interpreting competence dimensions covered in the published self-efficacy measures?

By answering these two research questions, the review aims to reveal the strengths and weaknesses of the measures reviewed, as well as potential measurement gaps that are yet to be addressed. Thus, it could reveal important implications for researchers interested in assessing these two constructs.

All the measures selected for review in this study were published in internationally recognized sources. To locate the published works in which these measures were included, we started first by searching for the relevant works in internationally well-known relevant journals, including *Across Languages & Cultures, Babel/International Journal of Translation, International Journal of Interpreter Education, Interpreting, Meta: Translators’ Journal, Perspectives, Target, The Interpreter & Translator Trainer, The Interpreters’ Newsletter, The Translator,* and *Translation & Interpreting Studies*. We searched for the works in these journals using keywords related to the two target constructs. The same keywords were also used in searches conducted through international databases and Google Scholar. This broader search revealed additional scales published in other multidisciplinary journals. These multiple search strategies helped us locate a total of seven published measures for the review (four translation self-efficacy and three interpreting self-efficacy scales). The seven scales were fully published by their developers in the works they authored. Furthermore, three additional studies reported using two interpreting self-efficacy scales (Cifuentes-Férez et al., 2024; Xu and Liu, 2023) and one translation self-efficacy scale (Yang et al., 2016), but the measures were not included in these studies and were therefore excluded from the review. We evaluated the seven published translation and interpreting self-efficacy measures in terms of their item representation of the assessed construct and the self-efficacy dimensions they covered.

## 4 Results of the review

The results of the review of the seven published translation and interpreting self-efficacy measures are given below. They are presented in two parts: the first covers translation self-efficacy measures, while the second addresses interpreting self-efficacy measures.

### 4.1 Published translation self-efficacy measures

The four translation self-efficacy measures reviewed were published in studies conducted by Stansfield et al. (1992), Bolaños-Medina and Núñez (2018), Konttinen (2021), and Yang et al. (2021). Table 1 provides a summary of these four measures.

**Table 1:** A summary of the reviewed translation self-efficacy measures.

Measure	Number of items	Overall focus
Stansfield et al.’s (1992) translation ability self-assessment scale	12 items	Rating one’s ability to perform some translation tasks
Bolaños-Medina and Núñez’s (2018) translation self-efficacy scale	20 items	Rating one’s ability to perform some skills related to pragmatic, strategic, self-evaluation and learning, problem-solving, and client-related competences of translation
Konttinen’s (2021) translation service provision self-efficacy scale	8 items	Rating one’s ability to perform some skills related to translation project management and translation production
Yang et al.’s (2021) translation self-efficacy	15 items	Rating one’s ability to perform some skills related to linguistic, extralinguistic, strategic, and psycho-physiological competences of translation

Stansfield et al. (1992) perhaps made one of the earliest attempts to develop a translation self-efficacy scale, which they labeled as ‘a translation ability self-assessment questionnaire.’ Their scale includes 12 items that assess respondents’ beliefs about their ability to perform some translation tasks, such as newspaper articles and editorials, policy and foreign diplomatic reports, scientific articles, correspondence, legal documents, evaluation reports, and training materials. This scale has a 4-point Likert response set—limited, functional, competent, and superior. Clearly, this is a simple scale with limitations, which can be attributed to the limited research on translation motivation during the early 1990s. The main limitation of Stansfield et al.’s (1992) measure is the absence of translation competences, as it merely assesses respondents’ perceived ability to perform a number of translation tasks. Another main limitation of the measure is the lack of validation data in the report published.

Bolaños-Medina and Núñez’s (2018) scale is perhaps the first published standardized translation self-efficacy measure. In its final version, the scale has 20 items with a 5-point Likert response set ranging from “cannot do at all” to “highly certain can do.” Experimenting with the measure on a sample of students, the authors conducted exploratory and confirmatory factor analyses, which showed that it has the following five components (four items each): (a) communicative/pragmatic competence (e.g., “*analyzing the skopos or the main function which will be required for a given TT*”), [sic] (b) self-evaluation and learning (e.g., “*recognizing translation mistakes as a whole*”), (c) problem-solving (e.g., “*generating different alternative solutions for translation problems*”), (d) client-related issues (e.g., “*appropriately dealing with the client during the whole process*”), and (e) strategic competence (e.g., *“adapting to the working conditions of every translation assignment in a flexible way*”) (pp. 67–68).

Another standardized scale was published by Konttinen (2021), who tried to assess students’ self-efficacy beliefs about performing some skills in translation company simulations. Konttinen validated the scale through a two-step confirmatory factor analysis. In the first step, it was found that the scale had 26 items loaded onto four factors related to translators’ strategic, operational, practical, and financial project management, as well as translation production abilities. The second step involved generating a more concise two-factor solution, with eight items loading onto two components: (a) translation project management (e.g., “*I am able to lead a translation organization*” and “*I am able to work as a project manager in translation project*”) and (b) translation production competences (e.g., “*I am able to revise translations in a translation project*” and “*I am able to assess the quality of a translated text*”), with four items for each component (p. 72). This scale has a 5-point response set ranging from “I strongly disagree” to “I strongly agree.”

Finally, Yang et al. (2021) validated a 15-item translation self-efficacy measure with a 5-point response set ranging from “I strongly disagree” to “I strongly agree.” Their factor analysis data revealed that the measure has the following three components: (a) translation (linguistic, extra-linguistic, and strategic) competence (n = nine items; e.g. “*I can translate the source text correctly and fluently*” and “*I can choose appropriate translation strategies in translation*”); (b) psycho-physiological competence (n = three items; e.g., “*I can improve my translation ability through continuous efforts*” and “*I can self-evaluate my translation integrity and appropriateness*”); and (c) external competence, which includes coping with difficulties, using digital resources, and translation service (n = three items; e.g., “*I can overcome difficulties in translation technology use*” and “*I can make full use of the digital resources to search information*”) (p. 7).

Compared to Stansfield et al.’s (1992) scale, the other three measures are more standardized. The developers of the three scales reported verifying their face and/or content validity and provided data indicating appropriate psychometric characteristics. In addition, these three measures cover a number of translation competences, including linguistic, extra-linguistic, pragmatic, and strategic competences, as well as those related to translation self-evaluation and learning, problem-solving, and translation project management. However, the three scales differ in the skills they assess within the competence dimensions they commonly address. For example, the items representing linguistic and strategic translation competences in the scales developed by Bolaños-Medina and Núñez (2018) and Yang et al. (2021) relate to different capabilities; the same applies to the items assessing translation project management in the three measures. Item representation in the last three measures is generally weak, as the statements included only capture limited dimensions of the target competences. All the above four measures were tested primarily with students rather than professional translators. The researchers in the last three studies reported generating item pools based on the conceptual analysis of the target competences and/or through literature reviews.

### 4.2 Published interpreting self-efficacy measures

The three interpreting self-efficacy measures reviewed were published by Jiménez Ivars et al. (2014), Lee (2014), and Chen (2018). Table 2 shows a summary of the three measures.

**Table 2:** A summary of the reviewed interpreting self-efficacy measures.

Measure	Number of items	Overall focus
Jiménez Ivars et al.’s (2014) interpreting self-efficacy scale	10 items	Rating one’s ability to solve interpreting problems and regulate interpreting problem-solving
Lee’s (2014) consecutive interpreting self-efficacy scale	21 items	Rating one’s ability to perform some consecutive interpreting skills and self-regulate interpreting learning, and their preference for interpreting task difficulty
Chen’s (2018) consecutive interpreting self-efficacy scale	11 items	Rating one’s ability to cope with difficulties in interpreting and complete interpreting tasks and beliefs about improving interpreting performance

Jiménez Ivars et al. (2014) adapted their interpreting self-efficacy scale from Baessler and Schwarzer’s (1996) general self-efficacy measure. The measure includes 10 items with a 7-point Likert response set ranging from “fully disagree” to “fully agree.” The 10 items generally focus on respondents’ perceived ability to solve interpreting problems and use strategies for overcoming them, for example:

*When interpreting…*

- *I can always manage to solve difficult interpreting problems if I try hard enough*.
- *I am confident that I could deal efficiently with unexpected events within an interpreting context*.
- *I can remain calm when facing difficulties because I can rely on my coping abilities (p. 186)*.

Jiménez Ivars et al. (2014) mentioned that the interpreting mode was not considered in their study, but they indicated that their student participants performed simultaneous and consecutive interpreting tasks as a part of the study. Similarly, they reported no validation data of their measure; only the reliability score was mentioned. Clearly, the measure has only one dimension: solving interpreting problems and regulating interpreting problem-solving.

Lee (2014) developed a 21-item consecutive interpreting self-efficacy scale with a 6-point Likert response set ranging from “very untrue of me” to “very true of me.” He generated the scale’s preliminary item pool (*n* = 63 statements) based on a review of literature on self-efficacy measures and interpreting competences; these statements were checked for their face and content validity. The exploratory factor analysis revealed that the 21 items loaded onto the following three components: (a) interpreting self-confidence (n = nine items; e.g., “*I’m confident that I can paraphrase B language speech accurately*” and “*I’m confident I can listen to a speech in an analytical way and deduce ideas from the context*”), (b) interpreting learning self-regulatory efficacy (n = four items; e.g., “*I can continue to make efforts to improve my interpreting memory*” and “*I can continue to make efforts to raise my summary and analysis capabilities*”), [sic] and (c) preference for task difficulty (n = eight items; e.g., “*I prefer an interpreting task in which word-for-word translation is sufficient*” and “*I dislike speech that requires a high degree of analytical thinking*”) (p. 198).

Another consecutive interpreting self-efficacy scale was published by Chen (2018), who developed its items by drawing upon literature reviews, expert interpreter interviews, and questionnaire data. The scale has 11 items loaded onto three factors: (a) coping with difficulties in interpreting (n = five items; e.g., “*I will not be impatient, and timely calm myself down when I find it’s difficult to interpret*” and “*I can always concentrate on finishing an Interpreting*”), [sic] (b) interpreting task completion (n = five items; e.g., “*I’m confident that I can better use all kinds of knowledge to grasp the meaning of the listening material and complete the language input*” and “*I’m confident that I can use my notes to help me finish the interpreting task*”), and (c) improving interpreting ability (n = one item; e.g., “*I believe I can improve my interpreting by working hard*”) (p. 846). A 5-point response set ranging from “completely disagree” to “totally agree” is used with the 11 items in the scale.

As may be noted, the above three self-efficacy scales cover only a limited number of interpreting competences, namely interpreting problem-solving, listening and production in interpreting task completion, and interpreting learning. This calls for addressing other interpreting competences in future measures. Similar to the reviewed translation measures, the three reviewed interpreting scales were also tested with students. In addition, two of these scales include some items unrelated to self-efficacy. Specifically, the 8-item third part of Lee’s (2014) measure does not assess respondents’ perceived ability to demonstrate particular interpreting competences, but rather their preference for interpreting task difficulty. Therefore, it assesses an attitudinal or dispositional dimension rather than a self-ability one. Unlike interpreting self-efficacy, which is a perceived ability construct, attitude toward interpreting reflects liking or disliking the situations in which one has to perform interpreting tasks. Similarly, Chen’s (2018) 11-item scale includes the following three items tapping into respondents’ interpreting giftedness and improvability beliefs:

- *I think I have a good mental quality and adaptability.*
- *I think I have a gift for English interpreting.*
- *I believe I can improve my interpreting by working hard (p. 846).*

Since these three items are about interpreting general ability, giftedness, and improvability beliefs, they are regarded as self-concept rather than self-efficacy statements (see Bong and Skaalvik, 2003; Dweck, 2000). In the general psychology literature, some other terms are used interchangeably with self-concept, such as the notion of giftedness, implicit theories of intelligence, the incremental theory of intelligence, and the entity theory of intelligence (see Abdel Latif, 2021). Regardless of such terminological differences, all these terms relate to individuals’ perceptions of their general ability in a given area, as well as their beliefs about the malleability and improvability of that ability, which differ from self-efficacy perceptions concerned with perceived task-specific ability beliefs. Given the nature of the attitude toward interpreting and interpreting self-concept, the measures developed by Lee (2014) and Chen (2018) are not pure measures of interpreting self-efficacy.

## 5 Conclusion

The above review indicates that the measurement of both translation and interpreting self-efficacy is still in its infancy. To date, only seven relevant measures have been published in the two fields. In both translation and interpreting self-efficacy measures, some competences are covered more than others, namely, linguistic, extra-linguistic, pragmatic, and strategic competences, as well as self-evaluation and learning, problem-solving, and project management, in translation self-efficacy measures, while problem-solving, listening and production, and learning in interpreting self-efficacy measures. Although a larger number of competences are covered in translation scales, there are other multiple translation and interpreting competences that are yet to be assessed in future self-efficacy measures in both fields. Competence representation is also problematic in the reviewed measures, as each translation or interpreting competence is represented by a limited number of items, not fully reflecting their sub-competences. In addition, two of the three reviewed interpreting scales are not pure self-efficacy measures as they include items representing attitudinal and self-concept dimensions. Overall, the above thorny issues imply the need for adopting different orientations in translation and interpreting self-efficacy measurement. Table 3 summarizes the self-efficacy measurement gaps.

**Table 3:** Some interpreting/translation self-efficacy measurement gaps need to be addressed in future research.

Interpreting/translation dimension	Self-efficacy measurement gaps
Genres	Developing measures covering different translation genres (e.g., legal, literary, and scientific translation) Building measures assessing medical, court, and conference interpreting Developing scales assessing simultaneous and sign language interpreting
Competences	Producing interpreting/translation textual features Using and regulating interpreting/translation strategies Performing interpreting/translation learning tasks Using interpreting−/translation-assisted technology Using project management tools
Item generation	Generating items by drawing upon interviews and/or questionnaire data Combining item interview/questionnaire data with construct conceptual analysis and literature reviews
Validation	Ensuring item face and content validity Ensuring items fully represent the target competence Examining construct validity via factor and/or Rasch analysis Verifying measure concurrent and/or predictive validity
Sampling	Collecting data from professional and trainee interpreters/translators Comparing both data types

Given the complexities of translation and interpreting task performance and the various genres that translators and interpreters deal with, we need multiple types of self-efficacy scales. For example, an important step toward advancing self-efficacy measurement in the two fields is developing measures that assess self-perceived abilities in specific areas such as legal, literary, and scientific translation, as well as medical, court, and conference interpreting. Similarly, the interpreting field still lacks self-efficacy measures for simultaneous and sign language interpreting. Particular translation and interpreting competence dimensions should be covered in separate self-efficacy measures, including the translation/interpreting product (the perceived ability to produce specific features in the target text), the translation/interpreting process [the perceived ability to implement cognitive acts of text conversion and regulate them; see, for example, Abdel Latif (2024)], translation/interpreting learning tasks, the use of translation/interpreting assistive technology, and project management.

Future measures pertaining to a given interpreting/translation competence should include an adequate number of items representing its skills more comprehensively. In this regard, generating items through construct conceptual analysis, literature review, and, more importantly, by collecting preliminary interview or questionnaire data could provide a richer item pool. Meanwhile, collecting preliminary data (i.e., for generating items) and main self-efficacy data from professional translators and interpreters will be beneficial for gaining deeper insights into performance difficulties related to the target task or genre. This step could help us identify a wider range of items representing the dimensions of competences. Combining data from professional interpreters and translators with data from students may inform us about the competence levels appropriate for interpreting/translation trainees versus professional interpreters/translators. Ultimately, we could have a variety of measures designed for assessing interpreting/translation self-efficacy in different genres at both the training and professional levels. The accuracy of the measure is also to be considered. For example, translation/interpreting self-efficacy items should not be conflated with items related to attitudes toward tasks or self-concept and its constructs (e.g., the notion of giftedness and theories of intelligence). Through appropriate face and content validity procedures, researchers can avoid such item and construct assessment conflation.

Some issues related to scale validation need to be considered. While factor analysis was used to identify the components of most reviewed scales, future researchers may test the construct validity of translation and interpreting self-efficacy measures using Rasch analysis, a more robust statistical tool (Hendriks et al., 2012). Combining Rasch analysis with factor analysis could also provide a comparative perspective on the construct validity of the measure. Since the response set may play a role in the predictive value of the self-efficacy measure (Pajares et al., 2001), future studies should examine the potential influence of different response sets in translation/interpreting self-efficacy measures. In line with Lee’s (2014) suggestion, future studies should also make use of criterion-related validity methods, such as concurrent validity—that is, correlating respondents’ scores on a self-efficacy scale and other measures—to validate their instruments.

Finally, due attention should be given to developing measures for assessing interpreting/translation self-concept scales. As indicated in the introduction, self-concept is closely related to self-efficacy but remains a distinct construct; therefore, its potential differentiating role is worth investigating. With the availability of measures assessing interpreting/translation self-concept beliefs, we could understand how these beliefs interact with interpreters’ and translators’ performance and work motives. Relevant self-concept measures could focus on dimensions such as the general interpreting/translation ability in various areas (e.g., I make a lot of mistakes in my interpreting/translation), the notion of giftedness (e.g., I am talented in interpreting/translation), and theories of interpreting/translation intelligence (e.g., my interpreting/translation quality will remain the same). For a comprehensive review of writing self-concept items that may be adapted for interpreting/translation self-concept measurement purposes, see Abdel Latif (2021). Needless to say, other motivational constructs within the interpreting and translation fields remain under-researched and consequently lack standardized measures. Future studies could focus on measuring and investigating under-explored constructs such as attitudes toward interpreting/translation tasks or professions, interpreting/translation anxiety, and learning goals. With these suggested studies, we can see future advancements in measuring interpreting/translation motivational constructs.
